# Mapping the miRNA landscape of primitive macrophage extracellular vesicles highlights their pro-vasculogenic effects in engineered human cardiac tissue

**DOI:** 10.1063/5.0313731

**Published:** 2026-04-07

**Authors:** Karl T. Wagner, Shira Landau, Gregory M. Kent, David F. Bodenstein, Milica Radisic

**Affiliations:** 1 Institute of Biomedical Engineering, University of Toronto, 27 King's College Circle, Toronto, Ontario M5S 1A1, Canada; 2 Department of Chemical Engineering and Applied Chemistry, University of Toronto, 27 King's College Circle, Toronto, Ontario M5S 1A1, Canada; 3 Toronto General Hospital Research Institute, University Health Network, 200 Elizabeth Street, Toronto, Ontario M5G 2C4, Canada; 4 Department of Medical Biophysics, University of Toronto, 101 College Street, Toronto, Ontario M5G 1L7, Canada; 5 McEwen Stem Cell Institute, University Health Network, 101 College Street, Toronto, Ontario M5G 1L7, Canada; 6 Department of Pharmacology and Toxicology, University of Toronto, Toronto, Ontario M5G 2C8, Canada; 7 Terrence Donnelly Centre for Cellular & Biomolecular Research, University of Toronto, 27 King's College Circle, Toronto, Ontario M5S 1A1, Canada

## Abstract

Resident cardiac macrophages, derived from primitive yolk sac precursors during embryogenesis, have increasingly been recognized for their distinct phenotype and functions in regulating homeostasis of the human heart. However, the profile of their extracellular vesicles (EVs) in cardiac signaling and regulation remains uncharted. Here, we employ differentiation of human pluripotent stem cell-derived primitive macrophages (Mac), harvesting their secreted EVs and performing in-depth characterization of associated microRNAs (miRNAs). Primitive macrophages secreted nanoscale EVs that expressed canonical EV markers, and miRNA sequencing highlighted a diverse and unique profile of miRNAs when compared to EVs sourced from other principal cardiac cell lineages and published data from monocyte-derived cells. In particular, we noted the abundance and enrichment of vascular-modulatory let-7 miRNAs and miR-126-3p. Functional screening of Mac-EVs in a 3D model of *in vitro* cardiac vasculogenesis confirmed enhanced early endothelial cell organization and branching. Establishing a reference for the human Mac-EV miRNome enables further hypothesis-driven mechanistic tests of Mac-EV miRNAs in mediating cardiac physiology and disease, opening the door to identification of therapeutic targets and modalities for cardiac repair.

## INTRODUCTION

Resident cardiac macrophages are important mediators of homeostasis in the adult human heart, yet their origin was only recently defined, and the understanding of their functionality is still emerging.^1–4^ Contrary to early theories postulating that tissue-resident macrophages arose solely from blood monocytes, it is now understood that most arise during early embryonic development and display distinct tissue-specific functions and phenotypes.^3,4^ Studies in rodent models have indicated that resident cardiac macrophages have roles in vascular development and angiogenesis, the cardiac electrical conduction system, and tissue recovery during and after injury.^1,5–10^ The physiological niche of resident macrophages in the human heart, however, remains poorly understood.^2^

Advances in human pluripotent stem cell (hPSC) differentiation have enabled the derivation of primitive human macrophages from CD43+ primitive hematopoietic progenitors, providing a source of cells that represent the yolk sac precursors that seed the developing heart during embryogenesis and give rise to resident cardiac macrophages.^1,11,12^ The availability of hPSC-derived primitive macrophages provides an experimentally accessible model to interrogate their physiological roles *in vitro*, addressing the difficulty in studying the functionality of similar cells in the native human heart *in vivo*. In a pair of recently published studies, 3D models of engineered heart tissue were successfully applied to demonstrate the utility of hPSC-derived primitive macrophages in enhancing the formation of stable and perfusable vasculature in cardiac tissues *in vitro*,^1^ and to uncover the beneficial role of primitive macrophages on the structural, phenotypic, and contractile maturation of cardiomyocytes (CMs).^2^ Despite these novel findings, the mechanisms of cell–cell communication between macrophages and other cardiac cell types in the human heart that mediate their physiological functions are still emerging, especially as it pertains to resident cardiac macrophage extracellular vesicle (EV) signaling.

Extracellular vesicles (EVs) are membrane-bound nano- and microparticles secreted by cells that can be taken up by nearby or remote recipient cells, often transferring biomolecular cargo or signals to elicit a biological response.^13,14^ microRNAs (miRNAs) present in the cargo of EVs have been a particular area of interest in studies of EV biology due to their relative enrichment in diverse EV populations and their distinct influence on cellular physiology via the RNA interference pathway.^14^ EVs secreted by cardiac cell types, including CMs, cardiac fibroblasts (CFs), and endothelial cells (ECs), have already demonstrated abilities to influence cardiac homeostasis, progression of cardiac disease, and even cardiac repair when applied as novel regenerative therapeutics.^13,14^ Observation of synergistic effects arising from heterogeneous miRNA-mediated signaling networks further supports holistic examination of EV-miRNAs from a broad spectrum of parent cell sources in tissues such as the heart.^15^

**FIG. 1.**
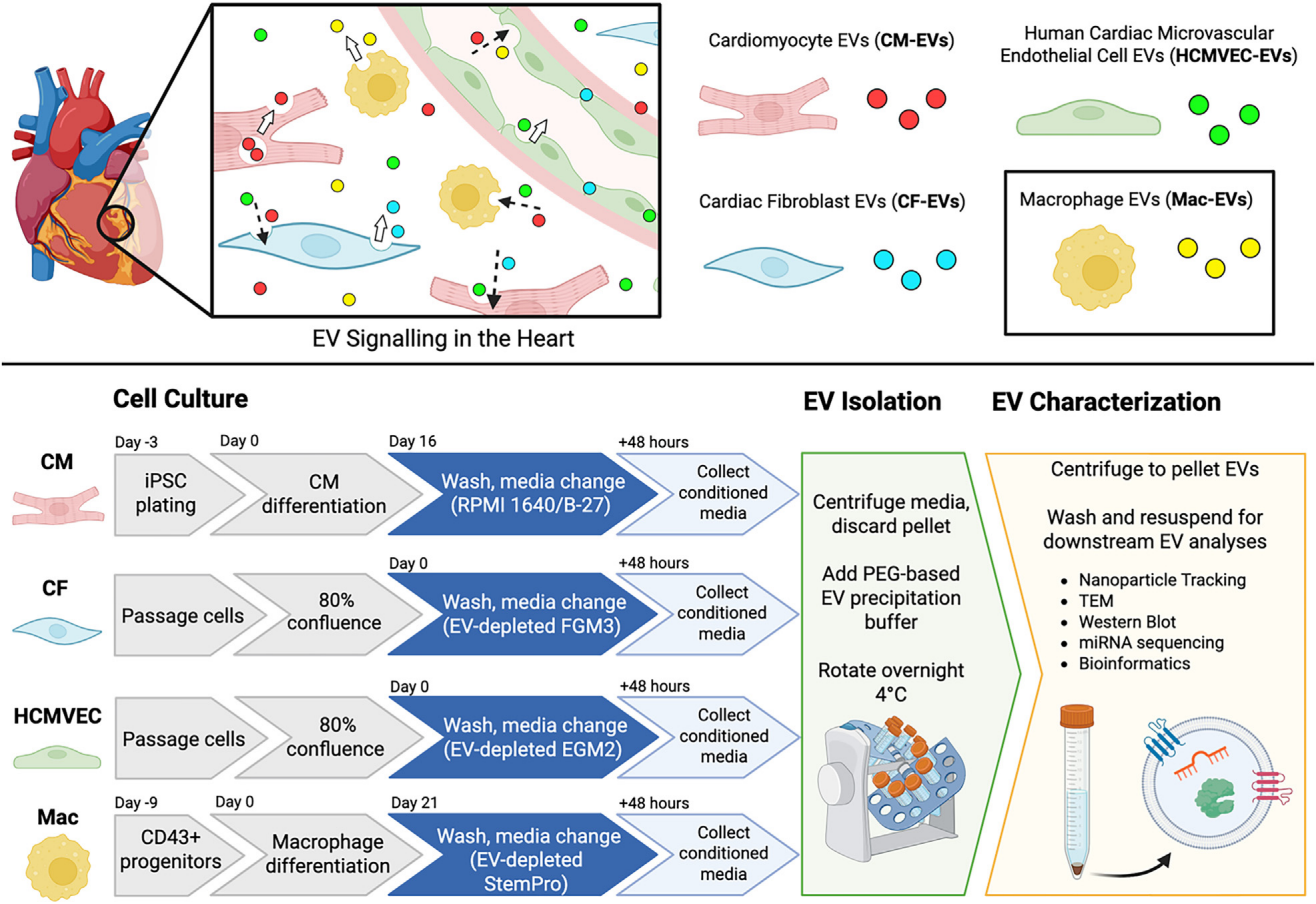
Experimental rationale and approach for mapping the landscape of macrophage and cardiac-EV-miRNAs in the context of *in vitro* heart tissue engineering. (a) Extracellular vesicle (EV) signaling between cardiac cells is postulated to play diverse and critical roles in mediating physiology, disease progression, and tissue repair in the heart. (b) In this study, EVs secreted by hPSC-derived primitive macrophages (Mac-EVs), as well as those derived from three principal cardiac cell types [induced pluripotent stem cell (iPSC)-derived cardiomyocytes (CM-EVs), primary cardiac fibroblasts (CF-EVs), and primary human cardiac microvascular endothelial cells (HCMVEC-EVs)] were separately concentrated from *in vitro* cell cultures for basic characterization toward mapping the landscape of cardiac-EVs and their miRNAs *in vitro*. Figure created using Biorender.

Increasing recognition of the distinct importance of resident cardiac macrophages in the physiology of the human heart motivates in-depth characterization of the profile of macrophage-secreted EVs (Mac-EVs) and their miRNA cargo to situate understanding of these cells within the greater cardiac-EV signaling milieu [Fig. 1(a)]. While numerous published studies have already isolated and characterized macrophage-secreted EVs, the EVs reported in the literature have primarily been sourced from rodent cells,^16–28^ human monocyte-derived cell lines (i.e., THP-1),^24,28–35^ or, less commonly, primary human monocyte-derived cells from plasma or peritoneal fluid.^36–38^ Consequently, the landscape of EV-miRNAs derived from primitive macrophages that have potential to acquire a tissue-resident phenotype^1,2,12^ remains uncharted, particularly as it relates to the signaling network of other cardiac cell-secreted EV subtypes and their miRNAs.

Here, we characterize the profile of hPSC-derived primitive macrophage-EVs and sequence their associated miRNAs. Using published literature for functional annotation of abundant and enriched Mac-EV miRNAs and contrasting their profile against other EV subtypes derived from principal cardiac cell lineages, we suggest cell-type specific roles of Mac-EVs in the heart and provide molecular context aligned with prior reports of macrophage-enhanced cardiac vasculogenesis in engineered heart tissues *in vitro*. This dataset provides a human-relevant reference for Mac-EV signaling in the heart, establishing a basis for further hypothesis-driven mechanistic tests and enabling future identification of novel EV therapeutic targets or modalities for treating heart disease.

## RESULTS

### hPSC-derived primitive macrophages release nanoscale EVs with canonical EV markers

We differentiated human primitive macrophages (Mac) prior to concentrating their secreted EVs, quantifying particle size distribution via nanoparticle tracking analysis (NTA), and employing transmission electron microscopy (TEM) and western blotting to obtain qualitative evidence of EV presence in our isolates. Previously established protocols were used for primitive macrophage differentiation from human embryonic stem cell (hESC)-derived hematopoietic progenitors.^1,11,12^ Flow cytometry indicated high expression (99%) of the canonical macrophage marker CD14 in our differentiation culture (supplementary material, Fig. S1).^1^

In parallel, we also separately established cultures of three other distinct cardiac cell types (representative of the most populous cell-type families present in the adult human heart^39^) for EV characterization: induced pluripotent stem cell (iPSC)-derived cardiomyocytes (CMs) were differentiated in monolayers,^40,41^ while primary human cardiac fibroblasts (CFs) and primary human cardiac microvascular endothelial cells (HCMVECs) were also seeded in separate flasks. Subsequent comparative profiling of EVs contextualized the signature of macrophage-EVs (Mac-EVs) among those derived from the other principal cardiac lineages.

**FIG. 2.**
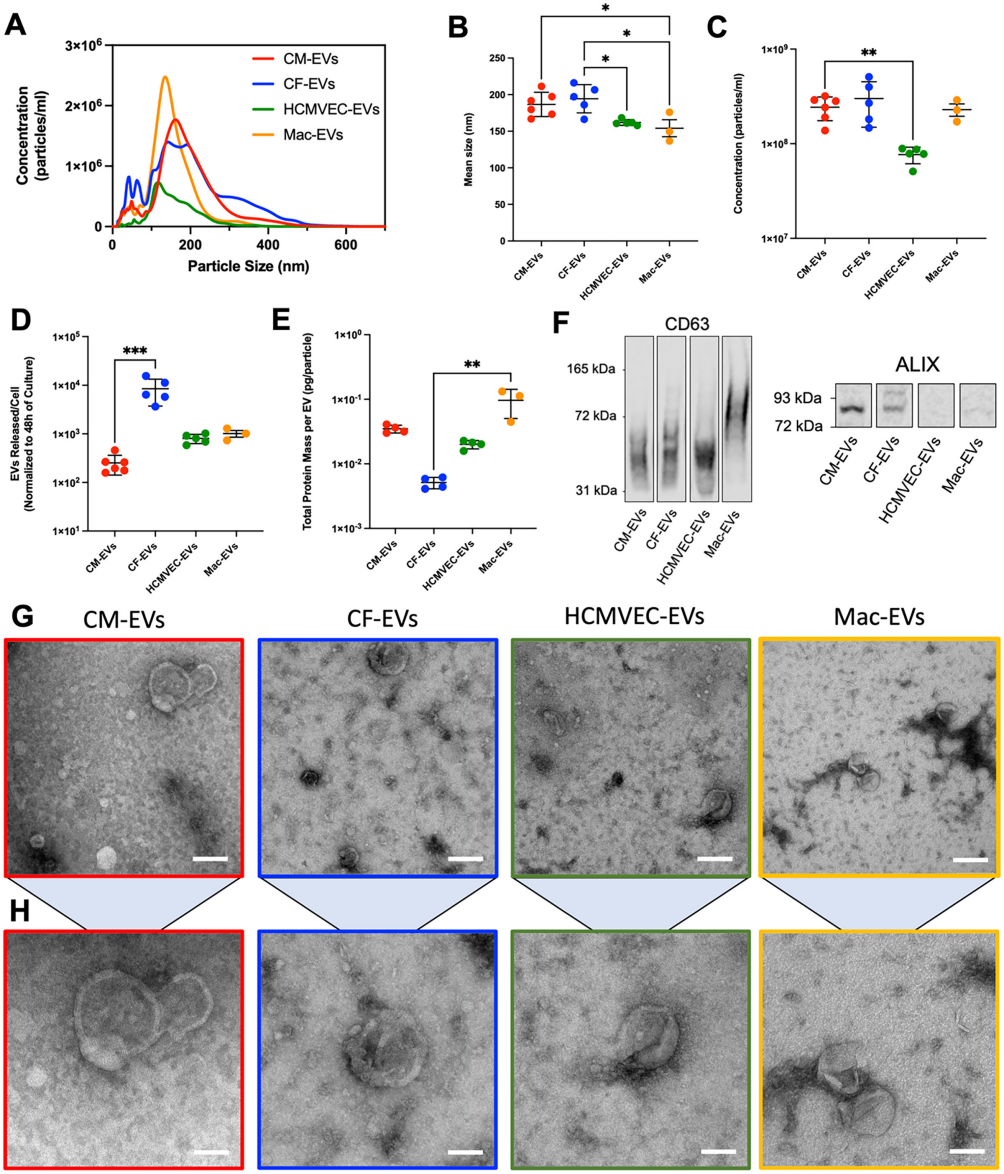
Comparative characterization of primitive macrophage-derived EVs with principal cardiac-EV lineages. (a)–(c) NTA-derived (a) average size distribution, (b) mean size, and (c) concentration of particles in cardiac-EV isolates. (d) Number of EV particles released per cell in cardiac cultures over a period of 48 h. (e) Total protein mass detected in EV lysates normalized to particle number. (f) Qualitative western blot detection of CD63 smear (non-reducing conditions) and ALIX (reducing conditions) in lysed EV isolates. (g) and (h) Representative TEM images of cardiac-EVs. (g) Scale bars: 200 nm, (h) scale bars: 100 nm. For (a)–(d): n = 6 for CM-EVs, n = 5 for CF- and HCMVEC-EVs, n = 3 for Mac-EVs. Data are presented as mean ± standard deviation (SD). For (b): One-way ANOVA with *post hoc* Tukey's multiple comparisons test; ^*^p < 0.05, ^**^p < 0.01. For (c): Brown–Forsythe and Welch ANOVA tests with *post hoc* Dunnett's T3 multiple comparisons tests with individual variances computed for each comparison; ^**^p < 0.01. For (d): Nonparametric Kruskal–Wallis test with Dunn's multiple comparisons test; ^***^p < 0.001. For (e): n = 4 for CM-, CF-, and HCMVEC-EVs, n = 3 for Mac-EVs. Nonparametric Kruskal–Wallis test with Dunn's multiple comparisons test; ^**^p < 0.01. Data are presented as mean ± SD.

At the differentiation end point, or when primary cell cultures reached 80% confluence, we replaced the media of each cardiac cell type with its appropriate formulation of “EV-depleted” media (where necessary) to remove the background contribution from media- or serum-derived EVs and then collected conditioned media after 48 h of culture for concentration of EV-containing preparations via PEG-based precipitation [Fig. 1(b)]. NTA revealed that the average size of particles harvested from all four cell types ranged from 154 nm for Mac-EVs to 194 nm for CF-EVs [Figs. 2(a) and 2(b)], with HCMVEC- and Mac-EV particles observed to be significantly smaller than those from CF-EV or both CM- and CF-EV samples, respectively. Average particle concentration for cardiac-EV isolates ranged from 7.66 × 10^7^ particles/ml for HCMVEC-EVs to 3.00 × 10^8^ particles/ml for CF-EVs [Fig. 2(c)], with Mac-EVs registering 2.29 × 10^8^ particles/ml on average. Significantly more particles were detected in CM-EV isolates compared to HCMVEC-EVs, while no significant differences in particle count were observed between CM-EVs, CF-EVs, and Mac-EVs. When normalized by cell number, CF-EV samples exhibited significantly more particles released per cell than CM-EV samples [Fig. 2(d)].

Following initial particle quantification of EV preparations, we performed lysis and protein quantification, finding that CF-EV isolates contained significantly less total protein per particle than Mac-EVs [Fig. 2(e)]. Qualitative western blotting positively detected the presence of the EV membrane marker CD63 and the intraluminal EV marker ALIX^42^ in isolates from each source of cardiac-EVs, though ALIX detection was noticeably faint in HCMVEC-EVs [Fig. 2(f); supplementary material, Fig. S2]. Variability in CD63 banding reflects expected cell-specific heterogeneity in CD63 glycosylation patterns,^43,44^ while the contributions of ALIX (a protein associated with endosomal sorting) to EV biogenesis have previously been shown to differ based on intracellular mechanisms of vesicle formation and release,^45^ possibly warranting future characterization of specific EV subtypes based on intracellular origin. TEM imaging identified EVs with a characteristic cup-shaped morphology in isolates from all cardiac cell types [Fig. 2(g)]. Overall, these data established a baseline biophysical profile of human primitive Mac-EVs within the cardiac-EV milieu, setting the stage for our primary analysis of the landscape of their associated miRNAs.

### Global miRNA sequence profiling defines both shared and lineage-enriched miRNAs in Mac-EVs

**FIG. 3.**
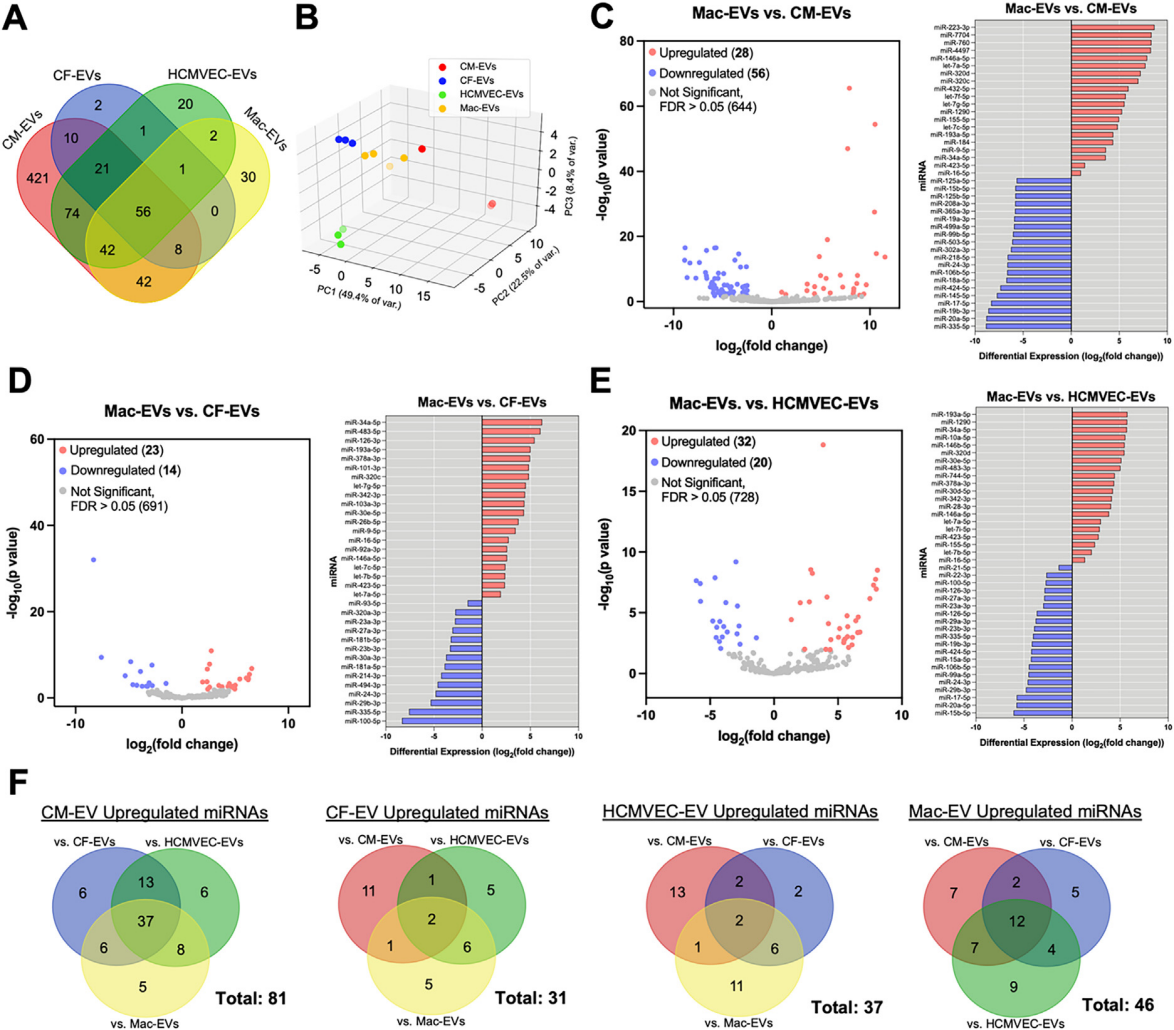
miRNA sequencing and differential expression analysis defined distinct and shared miRNAs expressed in Mac- and cardiac-EV preparations. (a) Venn diagram depicting distinct and overlapping miRNA species detected in sequenced EV samples. (b) 3D principal component analysis depicting clustering and separation between EV-miRNA samples by cell source. (c)–(e) Volcano plots alongside bar graphs depicting top up- and downregulated miRNAs for pairwise differential expression analysis comparisons between (c) Mac-EVs vs CM-EVs, (d) Mac-EVs vs CF-EVs, and (e) Mac-EVs vs HCMVEC-EVs. (f) Venn diagrams summarizing the total number of significantly upregulated miRNAs in each EV type vs every other cardiac-EV source. For (a)–(f): n = 3 for CM-, CF-, and HCMVEC-EVs; n = 4 for Mac-EVs. FDR < 0.05 is considered significant for differential expression analysis.

Following concentration and basic characterization of EVs, we performed next generation sequencing of miRNAs harvested separately from each of the four cardiac cell-type EV preparations to define the landscape of Mac-EV-associated miRNAs as compared to the three principal cardiac cell lineages (supplementary material Table 1). CM-EVs exhibited the most diverse range of miRNAs, with 674 different miRNA species detected in sequencing, 421 of which were exclusively detected in CM-derived EVs [Fig. 3(a)]. HCMVEC-EVs followed with 217 detected miRNAs; however, only 20 of these were unique to HCMVEC-EV isolates. Mac-EVs had the third largest panel of EV-miRNAs with 181 total species, but the second most unique miRNAs at 30. CF-EV samples contained both the fewest total detected miRNAs at 99 and the fewest uniquely detected miRNAs at 2. Of the 730 distinct miRNAs detected between all sources of cardiac-EVs, 56 were found in all four cardiac-EV types.

Principal component analysis (PCA) indicated distinct clustering and separation of EV-miRNAs by cell source, with CM-EVs separating to the greatest degree from the other cardiac cell types on PC1 (representing 49.4% of variance) [Fig. 3(b); supplementary material, Fig. S3]. Mac-EVs distinctly clustered on PC2 (representing 22.5% of variance), while PC3 was required to resolve the separation between HCMVEC- and CF-EVs (representing 8.4% of variance).

**FIG. 4.**
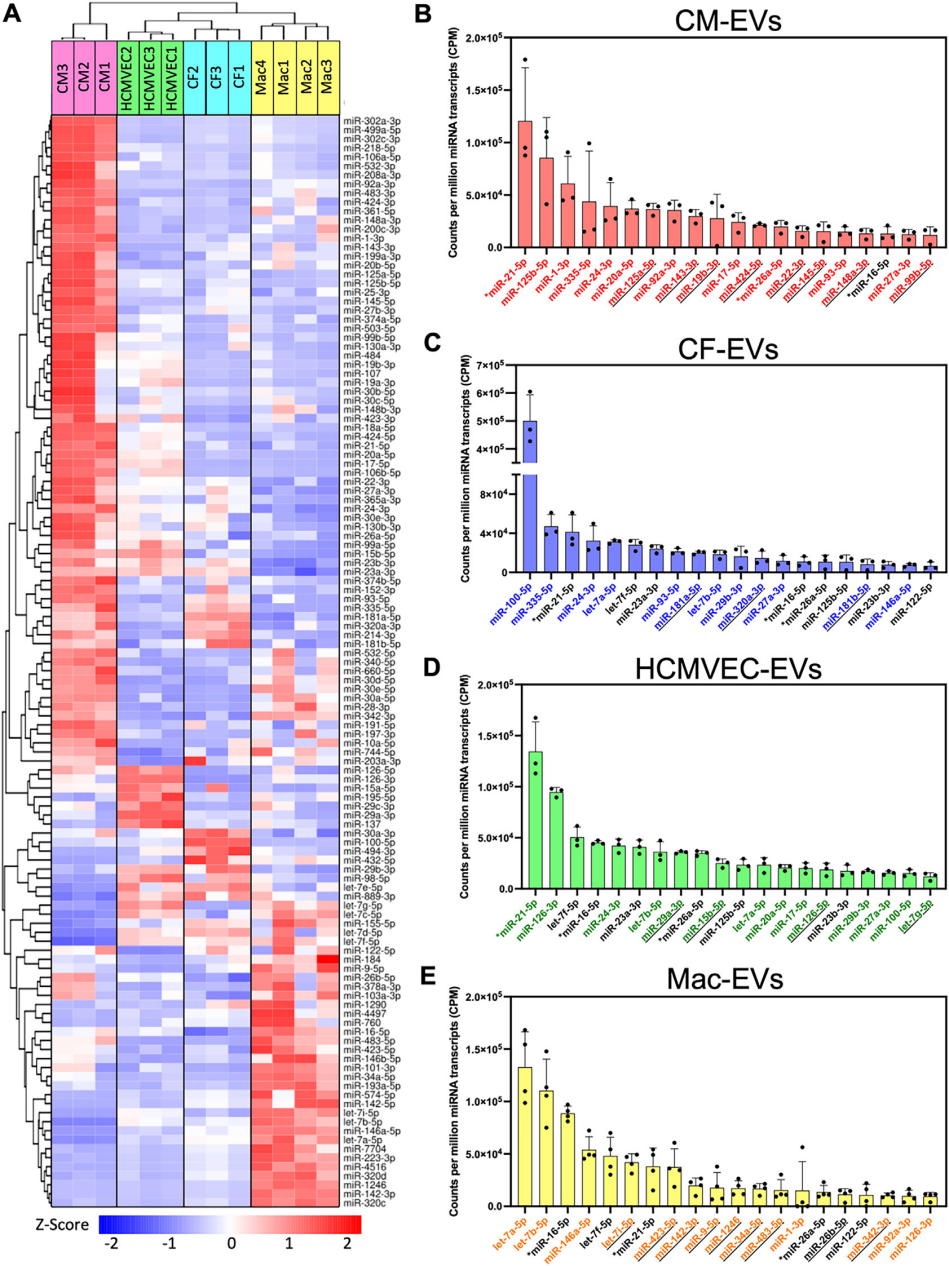
Comparative landscape of upregulated and abundant miRNAs detected in Mac-EV and cardiac-EV isolates. (a) Heatmap of all miRNAs significantly upregulated in at least one pairwise differential expression comparison between cardiac-EV subtypes. (b)–(e) Normalized counts (counts per million, CPM) of the top 20 most abundant miRNAs detected in (b) CM-EVs, (c) CF-EVs, (d) HCMVEC-EVs, and (e) Mac-EVs. ^*^ indicates miRNA is within the top 2 most abundant miRNAs for all four cardiac-EV subtypes. Underlined text denotes miRNAs that are unique to the top 20 most abundant miRNAs in a single EV subtype. Colored text highlights “key miRNAs” for a given EV subtype that were selected for downstream target prediction and pathway enrichment analyses based on three criteria: abundance, upregulation, and enrichment. For (a)–(e): n = 3 for CM-, CF-, and HCMVEC-EVs; and n = 4 for Mac-EVs. FDR < 0.05 is considered significant for differential expression analysis. Data are presented as mean ± standard deviation (SD).

Differential expression analysis was performed between each pair of cardiac-EV subtypes [Figs. 3(c)–3(f); and supplementary material Fig. S4 and Tables 2–7) to elucidate upregulated miRNAs based on EV source. Once again, CM-EVs exhibited the largest number of upregulated miRNAs, with 81 significantly upregulated in at least one pairwise comparison with the other three EV subtypes and 37 upregulated against all of the other EV types [Figs. 3(c), 3(f); supplementary material Figs. S4(a)–S4(d); and Table 8]. These included miR-145-5p and miR-19b-3p. Mac-EVs ranked second, with 46 miRNAs upregulated in at least one pairwise comparison and 12 upregulated against all three other EV sources [Figs. 3(c)–3(f); supplementary material Table 11]. Included in these 12 miRNAs commonly upregulated against all three principal cardiac lineage EV subtypes were three let-7 miRNAs (let-7a-5p, let-7b-5p, and let-7i-5p), miR-1246, miR-142-3p, miR-9-5p, and miR-146a-5p. HCMVEC-EVs possessed 37 total upregulated miRNAs, 2 of which were upregulated against all three other EV types: miR-126-3p and miR-29a-3p [Figs. 3(e), 3(f); and supplementary material Figs. S4(c)–S4(f) and Table 10]. CF-EVs had the fewest upregulated miRNAs with 31 total, 2 of which were upregulated against all three other EV types: miR-100-5p and miR-494-3p [Figs. 3(d) and 3(f); and supplementary material Figs. S4(a), S4(b), S4(e), and S4(f) and Table 9]. In total, 124 distinct miRNAs were differentially expressed between at least one pair of cardiac-EV subtypes [Fig. 4(a)].

### Integrative filtering highlights signature “key miRNAs” in Mac-EVs and cardiac-EVs

Following the analysis of upregulated EV-miRNAs, we turned our attention to the species that were most abundantly detected, highlighting the top 20 miRNAs with the highest number of normalized counts in each EV type [Figs. 4(b)–4(e)]. Three miRNAs were commonly found in the top 20 most abundant species of all four EV types [denoted by an ^*^ in Figs. 4(b)–4(e)]: miR-21-5p, miR-26a-5p, and miR-16-5p. miR-21-5p, in particular, was within the top three most abundant miRNA species detected in the three principal cardiac lineages (CM-EVs, CF-EVs, and HCMVEC-EVs) while ranking seventh in Mac-EVs.

For CM-EVs, miR-125b-5p and miR-1-3p followed miR-21-5p to round out the three most abundantly detected miRNAs [Fig. 4(b)]. Eight of the top 20 CM-EV-miRNAs were considered “uniquely abundant,” meaning they were not found within the top 20 of any of the other three cardiac-EV types [denoted by an underline in Fig. 4(b)]. These included miR-125a-5p, miR-143-3p, miR-19b-3p, miR-424-5p, miR-22-3p, miR-145-5p, miR-148a-3p, and miR-99b-5p. For CF-EVs, miR-100-5p leads the way in normalized counts by a noticeable margin, with miR-335-5p and miR-21-5p ranked next [Fig. 4(c)]. Three species were uniquely abundant in CF-EVs: miR-181a-5p, miR-320a-3p, and miR-181b-5p. HCMVEC-EVs were most abundant in miR-21-5p, miR-126-3p, and let-7f-5p and possessed four uniquely abundant species: miR-29a-3p, miR-15b-5p, miR-126-5p, and let-7g-5p [Fig. 4(d)].

Mac-EVs were most abundant in let-7a-5p, let-7b-5p, and miR-16-5p, while nine other miRNAs were considered uniquely abundant, the largest number when compared to any of the other three principal cardiac cell lineages [Fig. 4(e)]. These included let-7i-5p, miR-423-5p, miR-142-3p, miR-9-5p, miR-1246, miR-34a-5p, miR-483-5p, miR-26b-5p, and miR-342-3p. Notably, the vascular-modulatory let-7 family^46^ of miRNAs was represented in the top 20 miRNAs for all EV types except for CM-EVs, with four different let-7 species (including the top two spots) represented in this list for Mac-EVs, four for HCMVEC-EVs, and three for CF-EVs.

To further distill the broad cardiac-EV-miRNA landscape by lineage, we next applied an unbiased integrative filtering procedure to highlight signature “key miRNAs” for each EV type, prioritizing miRNAs that were hypothesized to most likely mediate EV functionality. Three criteria were used for this balanced identification of subtype-specific key EV-miRNAs: abundance, upregulation, and enrichment (supplementary material Fig. S5). To satisfy abundance and upregulation criteria, miRNAs that were both within the top 20 normalized counts [Figs. 4(b)–4(e) and supplementary material Table 1] and also upregulated in a given cardiac-EV subtype vs at least one other EV type [Fig. 4(a) and supplementary material Tables 8–11] were considered. For enrichment, we calculated the tissue-specificity index for each miRNA, Tau (τ) (supplementary material Table 12), a parameter widely used in transcriptomics to describe the tissue-specific enrichment of a given transcript on a scale from 0 (broadly expressed across groups) to 1 (specifically enriched in one group).^47,48^ To satisfactorily display cell-type enrichment, we required that an miRNA meet either a threshold of τ > 0.7 or fold change > 5 in at least 2 pairwise differential expression comparisons vs the other EV subtypes. Key miRNAs found to be abundant, upregulated, and enriched for each respective cardiac-EV subtype were highlighted with colored text in Figs. 4(b)–4(e). For Mac-EVs, these filtering criteria highlighted several additional EV-miRNAs of potential functional interest beyond those already identified, including miR-126-3p, miR-92a-3p, and miR-1-3p.

### Mac-EV exposure is associated with enhanced early vascular self-assembly in 3D *in vitro* cardiac tissues

After detecting prominent expression of potential vascular-modulating miRNAs in the Mac-EV miRNome, we interrogated the functional effects of Mac-EVs in a 3D fibrin hydrogel model of *in vitro* cardiac vasculogenesis.^1,49^ Although previous studies have found that direct cellular contact between macrophages and endothelial cells is required for longer term vascularization of *in vitro* cardiac tissue constructs,^1^ the contributions of the primitive macrophage secretome, including EVs, to the early stages of vascular self-assembly remain unclear.

**FIG. 5.**
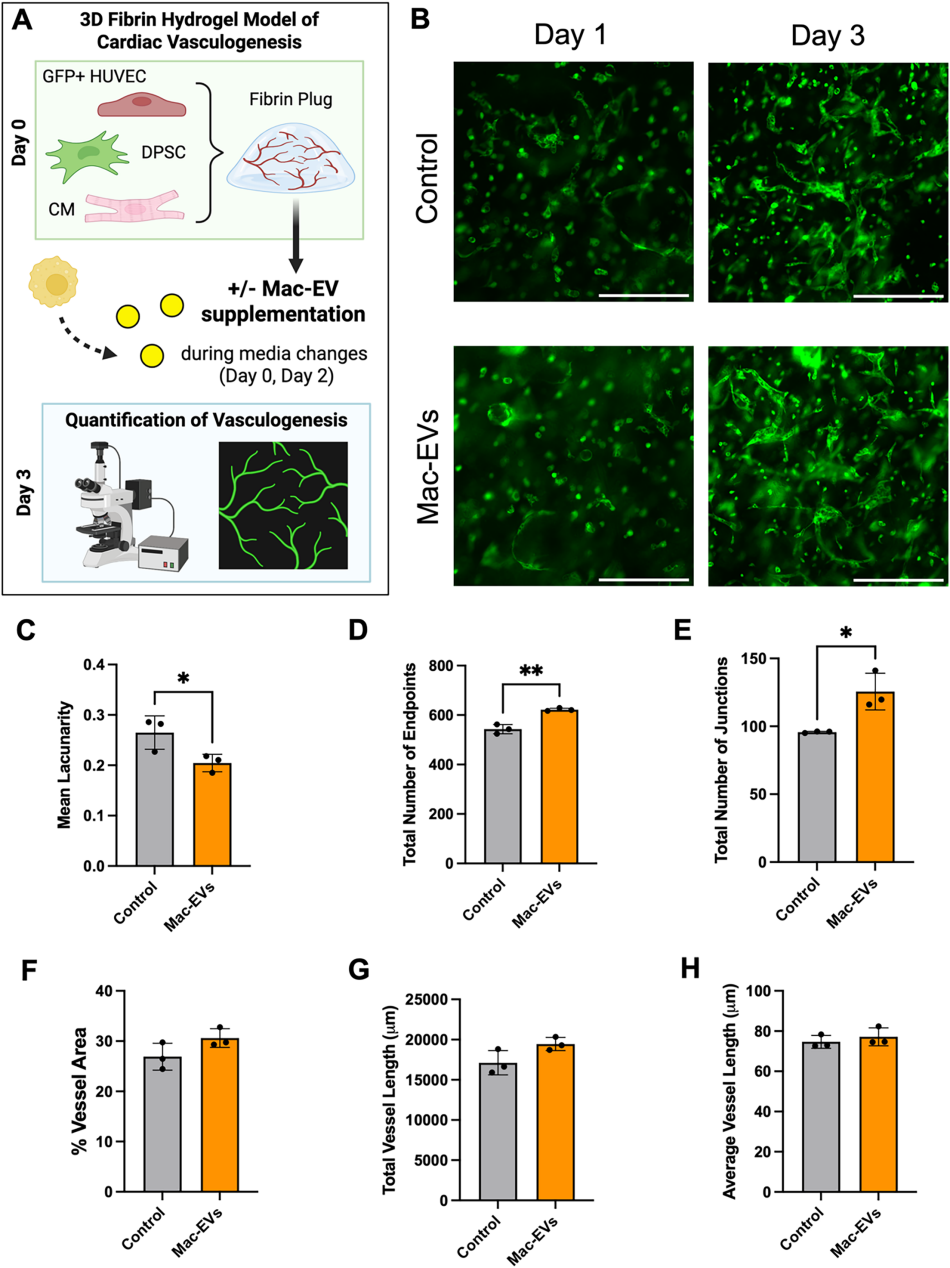
Macrophage-EVs enhance early vascular self-assembly in 3D *in vitro* cardiac tissues. (a) Schematic depicting the methodology for seeding 3D fibrin *in vitro* tissue models of cardiac vasculogenesis. GFP+ human umbilical vein endothelial cells (GFP+ HUVEC), dental pulp stem cells (DPSC), and cardiomyocytes (CM) were combined in previously optimized ratios within fibrin hydrogel plugs followed by supplementation of culture media with concentrated Mac-EVs and fluorescent quantification of HUVEC vessel formation. Figure created using Biorender. (b) Representative images depicting GFP+ HUVEC vessel formation from day 1 to day 3 after seeding for control tissues compared to tissues supplemented with Mac-EVs. Scale bar: 500 *μ*m. (c)–(e) Day 3 quantification of vasculogenesis in fibrin plugs with and without Mac-EV supplementation, comparing (c) mean lacunarity, (d) total number of endpoints, and (e) total number of junctions. For (c)–(e): n = 3 for control and Mac-EV groups. Data are presented as mean ± SD. For (c): unpaired t test, ^*^p < 0.05. For (d): lognormal t test, ^**^p < 0.01. For (e): lognormal Welch's t test, ^*^p < 0.05.

Applying a previously established and optimized model, we seeded 3D *in vitro* fibrin hydrogels with CMs, GFP+ human umbilical vein endothelial cells (HUVECs), and human dental pulp stem cells (DPSCs) as a stromal cell to support vessel formation.^1,49^ We supplemented tissue culture media with concentrated Mac-EVs and monitored the early impacts of EVs on GFP+ endothelial cell organization in tissue constructs via fluorescence microscopy [Figs. 5(a) and 5(b)]. After 3 days of culture, cardiac tissues supplemented with Mac-EVs exhibited a significant reduction in mean lacunarity, or “gapiness,” in self-assembling vessels [Fig. 5(c)] alongside a significant increase in total number of endpoints [Fig. 5(d)] and junctions [Fig. 5(e)]. Percent vessel area, total vessel length, and average vessel length did not differ significantly between groups (supplementary material Fig. S6).

## DISCUSSION

The existence of resident cardiac macrophages with a distinct origin and phenotype to those derived from circulating monocytes has been well documented.^3,4^ However, inherent challenges in accessing and studying native human heart tissue *in vivo*, alongside the limited availability of primary human cardiac macrophages for *in vitro* studies, have directly contributed to an underdeveloped understanding of the functional roles of these cells in the human heart, particularly as it pertains to Mac-EV signaling.^2^ While macrophage-derived EVs have already demonstrated impacts in cardiac disease progression,^16,24^ acute heart transplant rejection,^21^ and as potential pro-reparative cardiac therapeutics,^22,23,25,29,38^ current studies have primarily sourced EVs from animal cells or those derived from human primary or cell line monocytes. The derivation of primitive macrophages from hPSCs facilitates profiling of EVs and bioactive EV-miRNA cargo from macrophages that can acquire a tissue-resident phenotype while opening the door to future functional studies of Mac-EVs in 3D *in vitro* models of human heart tissue.^1,2^

Here, we detail the novel concentration and characterization of hPSC-derived primitive Mac-EVs. The current study highlights several distinct contributions. First, we situated the basic biophysical properties of Mac-EVs within the milieu of cardiac cell-secreted EVs. Second, we profiled the miRNome of Mac-EVs, using comparative analyses with other cardiac-EV subtypes alongside published literature to identify key Mac-EV miRNAs and connect these to hypothesized cardiac functions. Third, building on our findings from EV-miRNA sequencing as well as recent observations of primitive macrophage cells in the literature, we used a functional assay to demonstrate that Mac-EV exposure is associated with enhanced early vascular self-assembly in 3D engineered heart tissues *in vitro*. In producing and positioning this dataset of human primitive Mac-EV miRNAs, we open the door to further mechanistic studies of this sparsely explored EV subpopulation in cardiac physiology and pathophysiology that could enable the future identification of therapeutic targets or modalities.

Although particles in our concentrated Mac-EVs were slightly smaller in size, they were harvested in similar quantities to those from other principal cardiac cell-type lineages. The exception was CFs, which released the most particles per cell yet had significantly less total protein content in isolates compared to Mac-EVs. While our current study focused on profiling Mac-EV miRNAs, this finding raises questions regarding the extent to which protein sorting into secreted Mac-EVs may differ or impart functional consequences in a cardiac context, motivating future study of the Mac-EV proteome.

A similar pattern emerged in the context of miRNAs harvested from EV preparations, with Mac-EVs ranking second only to CM-EVs in both the number of unique and upregulated miRNA species, whereas CF-EVs again exhibiteithin the context of the heart provided std the lowest levels in both categories. The diversity and uniqueness of the Mac-EV miRNome wrong pretext for closer examination of their key miRNAs. miR-21-5p, miR-26a-5p, and miR-16-5p, commonly expressed in Mac-EVs and all three principal cardiac lineage EVs, have established links to physiological regulation in the heart. miR-21-5p, also found abundantly in human monocyte-derived macrophage-EVs and epicardial-EVs,^36,50^ has known ties to numerous cardiac regulatory roles including enhanced CM contractility^51^ and increased fibrosis in heart failure.^52^ miR-26a-5p has also been connected to cardiac-specific processes, including hypertrophy, fibrosis, and CM apoptosis.^53–57^ miR-16-5p has been detected with relative abundance in healthy human heart tissue^58,59^ and separately associated with CM stress and fibrotic CF activation.^60,61^

Several key miRNAs that were abundant and enriched in our primitive macrophage-EVs compared to the other cardiac-EV types were also conserved in published data for monocyte-derived macrophage-EVs and have been separately linked to physiological regulation in the heart. These included miR-146a-5p and miR-142-3p, which were found abundantly in monocyte-derived macrophage-EVs sourced from both classically activated/pro-inflammatory “M1” stimulated cells as well as alternatively activated/anti-inflammatory “M2” stimulated cells.^36^ miR-146a-5p has been contextually implicated in both cardioprotective effects, including improved cardiac function post-MI, and in promoting inflammation and cardiac cell dysfunction.^62–65^ miR-142-3p has similarly been linked to contextually differential regulation of cardiac hypertrophy, fibrosis, vasculature, and immunomodulation.^66–71^ A notable exception was miR-155-5p, which ranked 4th and 11th in abundance within published data from M1 and M2 monocyte-derived macrophage-EVs respectively,^36^ but fell outside the top 30 miRNAs in our primitive Mac-EVs. In a separate study, miR-155-5p-containing murine macrophage-EVs were found to increase inflammation in cardiac fibroblasts and increase the incidence of post-MI cardiac rupture in a mouse model of myocardial infarction (MI).

Conversely, we also noted a subset of key primitive Mac-EV miRNAs with expression profiles that distinguished them from both principal cardiac lineage EVs as well as published data from monocyte-derived macrophage-EVs, opening the door to further questions and future mechanistic investigations regarding the role of tissue-resident macrophage-EVs in human cardiac physiological regulation. The upregulated miR-483-5p, unique to the top 20 most abundant Mac-EV miRNAs, was previously reported as enriched in the mesoderm lineage of hESCs^72^ and also abundant in hESC-derived epicardial-EVs^50^ but was present at relatively low levels in human monocyte-derived macrophage-EVs.^36^ Similarly, miR-9-5p was enriched and abundant in our primitive Mac-EVs but was reported near or outside of the top 100 miRNAs in M1 and M2 monocyte-derived macrophage-EVs.^36^ While miR-9-5p has fewer well-documented roles in the heart, with some contradictory findings related to fibrosis modulation,^73,74^ cardioprotection,^75–77^ and heart failure progression,^74,78^ it was also highlighted as a key miRNA in recent characterization of epicardial-derived EVs post-cardiac ischemia reperfusion injury (IRI).^50^ miR-1246 was also noteworthy, being uniquely expressed in the top 20 Mac-EV miRNAs compared to each of the other cardiac-EV lineages and reported at relatively lower abundance in monocyte-derived macrophage-EVs.^36^ While miR-1246 has been shown to contribute to enhanced angiogenesis and reduced fibrosis *in vitro* and *in vivo* post-MI,^79,80^ more reports have focused on its role in the tumor microenvironment.^81^ Multiple studies have demonstrated that miR-1246 transferred from cancer cell-secreted EVs to macrophages induces M2 polarization to suppress inflammation in tumors,^82,83^ raising interesting questions related to potential effects in mediating resident cardiac macrophage immunomodulatory crosstalk in healthy vs diseased heart tissue.

The let-7 family was particularly prominent among key Mac-EV miRNAs, with let-7a-5p and let-7b-5p ranking as the top two species with the highest abundance in primitive Mac-EVs. Also abundant in HCMVEC-EVs and, to a lesser extent, in CF-EVs, let-7 miRNAs have been predominantly recognized for their regulation of vasculature *in vitro* and *in vivo*. Specifically, let-7s have shown functional roles in vascular formation, maintenance, barrier function, and dysregulation.^46^ Contradictory findings of both positive and negative regulatory roles of let-7a-5p and let-7b-5p on EC migration, tube formation, and viability have been linked to contextual and dose-dependencies.^49,84–88^ miR-126-3p, also identified as a key primitive Mac-EV miRNA ranking higher in relative abundance compared to reports in monocyte-derived macrophages,^36^ has similarly been linked to enhanced EC vascular tube formation.^89^ In previously published work, we showed that EVs derived from HUVECs were also enriched in let-7b-5p and miR-126-3p, and that transfection of cardiac cells with either miRNA alone enhanced vascular tube formation in cardiac tissues *in vitro*.^49^

Connecting these previous observations with similar key miRNAs highlighted in our Mac-EV sequencing data stimulated our subsequent functional investigation into the effects of Mac-EVs in modulating cardiac vasculogenesis *in vitro*. This investigation was also motivated by the fact that enhanced *in vitro* vasculogenesis represents one of the few cardiac-specific functions of macrophages exhibiting a tissue-resident phenotype that has been documented in recent literature,^1^ yet the specific contribution of secreted EVs to this process has not been defined. While direct cellular contact was identified as a key mechanism of macrophage-mediated vascular stabilization over longer timepoints, cardiac tissues cultured in a transwell setup with primitive macrophages exhibited similar vessel area to those cultured with direct cell mixing until day 4,^1^ prompting us to probe the short-term impacts of Mac-EVs on endothelial cell organization and vessel self-assembly in engineered cardiac tissues. Testing the supplementation of 3D cardiac tissue culture media with Mac-EVs to capture early effects on modulating vascular self-assembly revealed that even in the absence of macrophage cells, Mac-EVs significantly reduced vascular discontinuity and increased numbers of both endpoints and junctions in self-organizing ECs within 3D cardiac constructs. Together, these changes reflect enhanced endothelial cell organization and branching in tissues exposed to Mac-EVs, suggesting that Mac-EV-mediated signaling may contribute, at least in part, to early cardiac vascular formation *in vitro*.^1^

While we did not benchmark the vasculogenic activity of Mac-EVs against the other cardiac-EV lineages here, we previously published a study^49^ employing the same 3D fibrin hydrogel culture system and demonstrated CM-EV-mediated disruption of EC migration, proliferation, and vascular tube formation in contrast to EVs from human umbilical vein ECs, which enhanced cardiac vascular self-assembly. These findings are consistent with source-specific effects of EVs in mediating *in vitro* cardiac vascularization and warrant further investigation to define mechanisms of vascular regulation across EV lineages, particularly as it pertains to validating the activity of specific miRNAs. Our suggestion of a connection between Mac-EVs and pro-vasculogenic cardiac functionality aligns with mechanistic data that individually validated the pro-vasculogenic effects of let-7b-5p and miR-126-3p in the same culture system previously^49^ as well as previous observations of macrophage cell-mediated stabilization of cardiac vasculature *in vitro*,^1^ illustrating just one example of the numerous future opportunities for mechanistic studies of cardiac-specific functions of primitive Mac-EVs and their miRNA cargo that build upon the dataset that we provide here. Importantly, future work should also delineate whether different Mac-EV subpopulations carry distinct miRNA profiles that may underpin diverse functions of macrophage subtypes.

## LIMITATIONS AND FUTURE WORK

Here, we concentrated and characterized EVs from primitive macrophages, CMs, CFs, and HCMVECs, each cultured separately in isolation from one another. While this facilitated simple and reproducible harvesting of EVs with properties that could be directly attributed to their individual parent cell type, especially since precise cell-specific EV markers and isolation protocols for mixed EV populations have yet to be resolved,^90^ we also acknowledge the importance of context inherent to EV biology. Complex interactions and signaling between cell types within 3D native tissues can influence the biogenesis of secreted EVs as it pertains to active sorting of biomolecular cargo into EVs.^91^ With the construction of this dataset for primitive Mac-EVs, we establish a foundation for follow-up studies to validate mechanisms of Mac-EVs and the functionality of their individual key miRNAs in increasingly complex 3D engineered models of heart tissue *in vitro* and *in vivo*. Direct transfection of key Mac-EV miRNAs to cardiac cell and tissue cultures will be instrumental to this end.

While this study provides a comprehensive profile of Mac-EV-associated miRNA abundance, we acknowledge that the relatively modest miRNA sequencing sample sizes, despite being consistent with similar atlas-like characterization studies,^18,27,33,34,92–94^ may limit the robustness of extensive downstream functional inferences. Advanced miRNA target prediction and bioinformatic pathway enrichment analyses, techniques that could benefit from larger cohorts to support confident mechanistic interpretations, were not performed here but represent key tools that will help connect individual miRNAs with specific signaling pathways regulated by Mac-EVs within cardiac cells. To minimize potential platform-related bias, miRNA datasets generated from distinct sequencing and alignment workflows were harmonized before processing all samples within a unified downstream analytical framework. Results were interpreted conservatively and focused on defining the most highly abundant and consistently expressed EV-miRNAs, establishing an atlas-style dataset that can inform hypothesis-driven functional studies in future work.

In this study, we relied on a widely used approach,^92,95–99^ PEG-based precipitation, for global isolation of extracellular particles from the conditioned media of cardiac cell cultures. Though this provided an efficient, high-yield method for our broad and novel characterization of primitive Mac-EVs, it does not facilitate discrimination between specific particle subtypes based on intracellular mechanism of biogenesis or other non-membrane-bound material such as protein aggregates and ribonucleoprotein complexes that may be co-precipitated in PEG preparations. Future studies are warranted to build on our dataset here by delving further into the differences in proportion, miRNA profile, and functionality of various Mac-EV subtypes with distinct origins, such as exosomes arising from an endosomal origin vs microvesicles shed from cell membrane budding, as well as the contributions to such properties that can be attributed to non-membrane-bound co-precipitates. To this end, isolation of specific EV subpopulations using techniques such as size exclusion chromatography,^100,101^ density gradient separation,^102,103^ or immunoaffinity capture,^20,104^ alongside detergent sensitivity assays applying RNase or protease to EV preparations to assess contributions of protein aggregates and ribonucleoprotein complexes,^42,90^ represent important steps toward fully defining the profile of Mac-EVs and their roles in regulating cardiac physiology.

Beyond miRNAs, EV-associated proteins represent another class of biomolecular effector in EV-mediated signaling. While our study focused on profiling Mac-EV miRNAs and situating them within the context of the heart, we also emphasize that future studies characterizing the Mac-EV proteome are also essential to building a more complete picture of the role and mechanisms of Mac-EVs in the human heart.

## CONCLUSION

In this study, we demonstrated that hPSC-derived primitive macrophages secrete nanoscale EVs with a distinct biophysical and miRNA profile within the context of EVs derived from principal cardiac cell lineages. Next generation sequencing highlighted the diverse and abundant miRNA species in Mac-EV preparations. Differential comparisons with respect to CM-EVs, CF-EVs, and HCMVEC-EVs facilitated further identification of unique, enriched, and upregulated miRNAs in Mac-EVs. Cross-referencing our findings with published data emphasized the unique signature of primitive Mac-EV miRNAs, including miR-483-5p, miR-9-5p, and miR-1246, when compared to those isolated from monocyte-derived macrophage-EVs, the current paradigm in the literature. In particular, the prominence of let-7 miRNAs and miR-126-3p in Mac-EVs provided substance for a connection between previous studies separately showing pro-vasculogenic effects of these specific miRNAs and of primitive macrophage cells in cardiac tissues. Aligning with these findings, we observed an association between Mac-EV exposure and enhanced cardiac vascular self-assembly *in vitro*. By characterizing EVs from hPSC-derived primitive macrophages in this study, we underlined the distinct EV profile of macrophages with a more representative tissue-resident phenotype and situated the cardiac-specific relevance of the Mac-EV miRNome, highlighting the potential functional relevance of Mac-EVs to cardiac vascular formation. Establishing a reference for Mac-EV signaling in the human heart creates a foundation for future studies that can validate specific mechanisms of Mac-EV miRNAs in regulating cardiac physiology and disease, ultimately opening the door to the identification of therapeutic targets and opportunities related to Mac-EVs and their miRNAs.

## METHODS

### Cell culture and differentiation

Primitive macrophages: the H1 hESC line was differentiated into CD43+ primitive hematopoietic progenitors according to a published protocol,^11,12^ which were isolated via magnetic-activated cell sorting at day 9 of differentiation and cryopreserved. To complete macrophage differentiation, cells were thawed then cultured in suspension for 3 days in poly-HEMA coated 6 well plates at approximately 500 000 cells per well in supplemented StemPro-34 base medium (2 ml per well, containing transferrin (ROCHE 10652202001, 150 *μ*g/ml), ascorbic acid (Sigma Aldrich A4544, 50 *μ*g/ml), L-glutamine (ThermoFisher 25040081, 2 mM), and monothioglycerol (Sigma Aldrich M6145, 50 *μ*g/ml) with the addition of MCSF (R&D Systems 216-MC, 30 ng/ml), IL3 (R&D Systems 203IL, 50 ng/ml), and SCF (R&D Systems 255SC, 100 ng/ml). Then, for 18+ days, cells were cultured in the same supplemented StemPro-34 base medium with the addition of MCSF (30 ng/ml) along with media changes performed every 3–4 days.^1,12^

CMs were differentiated from BJ1D human iPSCs as described.^1,40,49^ Briefly, iPSCs cultured in mTeSR Plus medium (STEMCELL Technologies) were passaged at 0.75 × 10∧6 cells/well onto 12-well Matrigel (Corning) coated plates. When confluent, the medium was changed to RPMI supplemented with B27 minus insulin (Gibco), 1% Pen-Strep, and 8 *μ*M CHIR99021 (Cayman Chemical) for 24 h. On day 3, medium was changed to the same RPMI based medium (without CHIR) plus 5 *μ*M IWP4 (Stemgent) for 48 h. Cells were then cultured in RPMI with B27 minus insulin until day 7, then RPMI with B27 supplement (Gibco) thereafter. After at least day 14, CMs were harvested by 30 min treatment with 10x TrypLE Express (Gibco) at 37 °C, media was added to neutralize TrypLE and rinse wells, then cells were centrifuged for 5 min at 300 g, resuspended in medium, and live cells counted with addition of trypan blue.

Human ventricular cardiac fibroblasts (CFs) (Lonza, CC-2904) were cultured in Fibroblast Growth Medium 3 (FGM3; PromoCell, C-23025) with 1% 10 000 U/ml penicillin/streptomycin in T75 or T175 flasks.

Human cardiac microvascular endothelial cells (HCMVECs) (Applied Biological Materials Inc, T0514) were cultured in Endothelial Cell Growth Medium 2 (EGM2; PromoCell, C-22011) with 1% 10 000 U/ml penicillin/streptomycin in T75 or T175 flasks.

### Macrophage flow cytometry

Flow cytometry to quantify the proportion of CD14+ Mac cells was performed as previously described.^1,2^ Briefly, differentiated Mac were stained with mouse anti-human CD14 (BioLegend 301814; 1:100) for 20 min at 4 °C. Cells were washed in fluorescence-activated cell sorting (FACS) buffer consisting of 2% heat-inactivated bovine serum in PBS with 1 mM EDTA., centrifuged for 5 min at 300 g and 4 °C, resuspended in FACS buffer, filtered (40 *μ*m), then quantified on a BD LSRFortessa flow cytometer. Data were analyzed on FlowJo (v10.10) compared to an unstained control.

### Conditioned media collection

Following Mac differentiation, supplemented StemPro-34 base medium with the addition of MCSF (30 ng/ml) was prepared as before then depleted of background particles via ultrafiltration: media was centrifuged in Amicon Ultra-15 100 kDa centrifugal filters (MilliporeSigma, UFC910024) at 4000 g for 30 min at room temperature. Mac cells in suspension were pelleted via centrifugation at 200 g for 5 min, supernatant was removed, and cells were resuspended in 1 ml of medium for counting in the presence of trypan blue. Cells were then centrifuged and resuspended in 4 ml ultrafiltered medium per well of cells collected from a 6 well plate. After 48 h, conditioned media were collected and centrifuged to pellet cells. The supernatant was frozen at −20 °C until EV concentration.

Following CM differentiation, between days 14–22, conditioned media (RPMI with B27 supplement) was collected from plates after 72 h of culture (in accordance with the regular media change timeline for the CM differentiation protocol) until CMs were dissociated and counted. Conditioned media were frozen at −20 °C until EV concentration.

For CFs, when cells reached 80% confluence, flasks were washed with PBS, then media were changed to un-supplemented basal FGM3 with the addition of 10% exosome-depleted FBS (ThermoFisher Scientific, A2720803) and 1% 10 000 U/ml penicillin/streptomycin. 15 ml of media was used in T75 flasks and 35 ml in T175 flasks. After 48 h of culture, conditioned media were collected and frozen at −20 °C until EV concentration. Cells were detached from flasks with trypsin, centrifuged, and resuspended for counting in the presence of trypan blue. HCMVEC conditioned media were collected using the same protocol as CFs except that un-supplemented basal EGM2 with the addition of 2% exosome-depleted FBS and 1% 10 000 U/ml penicillin/streptomycin was employed.

### EV concentration

Conditioned media collected separately from each cell type were thawed prior to EV concentration as described previously.^49,50^ Briefly, conditioned media were centrifuged for 10 min at 3200 g to pellet debris. The supernatant was collected, followed by the addition of either polyethylene glycol (PEG) 6000 buffer^49,96^ (0.4 ml per 1 ml conditioned media, final concentration of 8% w/v PEG) or the PEG-based miRCURY exosome isolation kit (Qiagen, 76743; same volume as PEG 6000, applied for EV prep before miRNA sequencing). After rotation overnight at 4 °C, EVs were pelleted from PEG-media solution via centrifugation at 3200 g for 30 min at 21 °C. PBS was used to rinse pellets without resuspension, then pellets were resuspended in Resuspension Buffer (Qiagen, 76743) using a volume equal to 1/100th of the input conditioned media volume, producing a preparation of EVs that was effectively 100× concentrated relative to the initial conditioned media sample (referred to hereafter as “100× concentrated EVs”) before storage at −80 °C. For western blotting and miRNA sequencing, pellets were resuspended directly in RIPA or Qiazol lysis buffers, respectively, as outlined previously.^49^

### Nanoparticle tracking analysis

10 *μ*l aliquots of 100× concentrated EVs (prepared as described above) were diluted to 1x in 990 *μ*l of PBS for quantification of size distribution and concentration via nanoparticle tracking analysis (NTA). Samples were briefly vortexed and mixed with a pipette before being drawn into a sterile 1 ml syringe. NTA was performed on a Nanosight NS300 (Malvern). The NTA flow cell was washed with 1 ml of PBS prior to insertion of 1× EV sample, with manual flushing of at least 300 *μ*l sample through the flow cell prior to setup on a syringe pump. The syringe pump was set to 60, and the focus plane was adjusted to the point where the maximum number of particles were in focus. A base camera level of 13 and a gain of 3 were used, with fine tuning to achieve a level just below pixel saturation. Three 30 s captures were taken for each sample and analyzed at a threshold between 5 and 6, with results averaged together for a single biological replicate as appropriate. Between samples, the NTA flow cell was flushed with 1 ml of PBS. Where indicated, NTA particle concentrations were scaled to the amount of input conditioned media, then normalized to the number of cells counted in a given sample and to a 48 h culture period where required (for CM-EVs).

For particle normalization to total protein mass, concentrated EV isolates were prepared as described above from aliquots of between 3.5 and 5 ml of conditioned media. NTA samples were prepared and quantified, then the remainder of the 100× concentrated EV preparations were each separately diluted with 50 *μ*l of RIPA buffer for EV lysis, mixed thoroughly with a pipette, then vortexed briefly. The Pierce BCA Protein Assay Kit (ThermoFisher Scientific, 23225) was used to quantify total protein mass according to the manufacturer's protocols using an input of 10 *μ*l for samples/standards in the 96 well plate assay format.

### Western blotting

EV lysis, total protein quantification, and qualitative western blotting for CD63 and ALIX were performed as outlined previously.^49^ Briefly, pelleted EVs were lysed directly in 100–120 *μ*l of RIPA buffer and vortexed. The total protein concentration was quantified using the BCA assay as described above. 8 *μ*l of each sample was used to load gels, except for Mac-EVs, where 3.7 *μ*g of the sample was used due to the limited availability of macrophages/conditioned media. For ALIX blots, samples were mixed separately with sample loading buffer, reducing agent, and water and then incubated for 10 min at 70 °C as described (reducing conditions).^49^ For CD63 blots, samples were mixed with sample loading buffer and water and then incubated at room temperature for 20 min (non-reducing conditions). Samples were centrifuged, loaded into gels, and subjected to electrophoresis as outlined,^49^ except for the use of MES SDS running buffer (Invitrogen) here. After electrophoresis, gels were rinsed, transferred to PVDF membranes using an iBlot dry transfer device (Invitrogen), and the REVERT total protein stain (LI-COR) was performed and imaged on an Odyssey Fc imager (LI-COR). Following the published protocol,^49^ membranes were blocked with skim milk buffer, and primary staining was performed overnight at 4 °C (mouse anti-CD63, Abcam, ab271286, 1:1000, non-reducing blots; rabbit anti-ALIX, ThermoFisher Scientific, MA5- 32773, 1:1000, reducing blots). Blots were washed, secondary staining was performed for 2 h at room temperature (anti-mouse IRDye800CW, LI-COR, 925-32212, 1:10 000; anti-rabbit IRDye800CW, LI-COR, 925-32213, 1:10 000), and blots were washed again, then imaging was performed on the Odyssey Fc.^49^

### Transmission electron microscopy

TEM imaging of EVs was performed as described previously.^49^ Briefly, 5 *μ*l of 100× concentrated EV samples from CMs, CFs, or HCMVECs were pipetted separately onto plasma treated carbon-coated copper TEM grids (Electron Microscopy Sciences). After a 1 min incubation, the sample was wicked with wet filter paper, washed with 5 *μ*l DI water, wicked again, then stained with 2% uranyl acetate (Electron Microscopy Sicences) for 30 s before a final wicking. Grids were imaged on a Talos L120C TEM (ThermoFisher Scientific) at 45 000×. For Mac-EVs, a 30 *μ*l droplet of 100× concentrated EV sample was deposited onto parafilm, and a plasma treated TEM grid was placed on the droplet for 1 min. The grid was removed, then placed onto a droplet of DI water briefly for washing, placed onto a droplet of 2% uranyl acetate for 30 s, and then removed and imaged on an HT7800 TEM (Hitachi) at 45 000×.

### miRNA sequencing

The miRNeasy Micro Kit (Qiagen, 217084) was used to isolate miRNA directly from pelleted EV preparations according to the manufacturer's protocols, as previously described.^49^ For CM-, CF-, and HCMVEC-EV samples, miRNA enrichment and DNase treatment steps were performed as part of the prescribed protocol, miRNA quantification and sequencing were performed at the Donnelly Sequencing Center, and library preparation and alignment were performed using the Bio-Rad SEQuoia kit and SeqSense software as published.^49^ For Mac-EVs, isolated miRNA was sent to Novogene for DNase treatment, quantification, library preparation, sequencing, and trimming as described previously, with the Qiagen RNA-seq Analysis Portal 3.0 (workflow version 1.2) applied for alignment and counting.^97^ Count file format was harmonized between the alignment pipelines for compatibility with downstream analyses; miRNAs absent in one pipeline with non-zero counts in the other were manually added as zero count entries where relevant, while miRNAs missing -3p/-5p delineation in the BioRad pipeline were determined based on the solely expressed form in the Qiagen pipeline as well as the provided chromosome location. EV-miRNA sequencing data were deposited in the GEO repository (accession: GSE311002).

Principal component analysis (PCA) was performed using the PCAGO^105^ web tool, with DESeq2 rlog transformation for normalization, then plotted with SRPlot.^106^ Differential expression analysis was performed using DESeq2 on the Galaxy web platform.^107,108^ All samples/groups were included in one simultaneous DESeq2 analysis with all groups compared against every other group and outlier filtering disabled. FDR-adjusted p value < 0.05 was considered significant. miRNA species significantly different between one or more pairwise comparisons between groups were plotted in a heatmap on SRPlot using rlog normalized counts from DESeq2 analysis. Raw miRNA counts were normalized to the total number of reads and then converted to counts per × 10∧6 miRNA transcripts for each sample. Tissue-specificity index (τ) for each miRNA was calculated from average DESeq2 normalized counts for each EV subtype according to a defined formula.^47,48^

### Mac-EV cardiac vasculogenesis assay

GFP+ HUVECs (Angio-Proteomie) were cultured in EGM2, while DPSCs (Lonza) were cultured in in low-glucose DMEM with the addition of 10% FBS, 1% NEAA, 1% GlutaMAX, and 1% Pen-Strep. Media were changed every 2 days. Prior to tissue seeding, cells were treated with 0.05% trypsin for 5 min at 37 °C, neutralized with media, centrifuged for 5 min at 300 g, then counted. iPS-CMs were differentiated, harvested, and counted as outlined earlier.

3D fibrin hydrogel cardiac tissues were formed as described previously.^1^ Briefly, resuspended and counted HUVECs/DPSCs/CMs were mixed at a previously optimized ratio^1^ of 25:25:50 and then centrifuged for 5 min at 300 g with careful removal of media from cell pellet. A total cell concentration (for all three cell types combined) of 6.6 × 10^6^ cells/ml was used to form tissues (each tissue having a final total volume of 12 *μ*l). The mixed cell pellet was resuspended in 9 *μ*l of human fibrinogen (30 mg/ml, Sigma-Aldrich) per tissue to be seeded, pipetted thoroughly, then mixed with 3 *μ*l of human thrombin (33 U/ml, Sigma-Aldrich) per tissue and kept on ice. 12 *μ*l of the cell-gel mixture was quickly pipetted into the middle of the well on a 12 well plate and incubated for 30 min at 37 °C before the gentle addition of 500 *μ*l of culture medium per well. The medium consisted of 50% EGM2 and 50% I3M [supplemented StemPro-34 with 1% pen/strep, 213 *μ*g/ml 2-phospho-L-ascorbic acid trisodium salt (MilliporeSigma, 49752), 150 *μ*g/ml transferrin (MilliporeSigma, T8158), 20 mM HEPES, and 1%GlutaMAX]. For Mac-EV supplemented tissues, 20 *μ*l of 100× concentrated Mac-EVs was added to each well along with 500 *μ*l of media after seeding. Media were changed on day 2 (with or without the addition of supplemental Mac-EVs).

GFP+ HUVECs were imaged and subjected to Angiotool^109^ quantification of vascularization as previously outlined.^49^ Briefly, tissues were imaged at 10× on an Olympus IX81 fluorescent microscope in the FITC channel on days 1 and 3 to track early changes in HUVEC organization, focusing on the vertical plane in the middle of 3D tissues with the majority of cells in focus and using consistent exposure and laser power. Day 3 image brightness was automatically adjusted in ImageJ^110^ then loaded into Angiotool^109^ for quantification with the following settings: threshold 12–255; thickness 15; remove small particles 200; and fill holes 1500.

### Data and statistical analyses

Unless indicated, statistical analyses and plotting were performed using GraphPad Prism (v10.5.0). Data are presented as mean ± standard deviation (SD). Sample sizes are included in figure captions. For NTA, ordinary one-way ANOVA with a *post hoc* Tukey test for multiple comparisons was used when the Shapiro–Wilk test for normality passed and the Brown–Forsythe test did not indicate a difference in SDs. Where normality did not pass, the Kruskal–Wallis nonparametric test was used with the *post hoc* Dunn's multiple comparisons test as indicated in the captions. Where normality passed but SDs were significantly different, the Brown–Forsythe and Welch ANOVA tests were used with Dunnett's T3 multiple comparisons tests with individual variances computed for each comparison as indicated in captions. For Angiotool quantification, parameters were tested for lognormal distribution to account for the multiplicative nature of vessel formation and assembly, except for lacunarity, which was tested for normality since it represents a dimensionless index. Where the Shapiro–Wilk normality test passed (lacunarity) and SDs were not significantly different via the F test, data were analyzed using an unpaired t test. Where the Shapiro–Wilk lognormality test passed and SDs were not significantly different via the F test, data were analyzed using a lognormal t test. Where SDs were different, the lognormal Welch's t test was used. Where lognormality did not pass, the nonparametric Mann–Whitney test was used. p < 0.05 or FDR-adjusted p < 0.05 was considered significant where appropriate, as indicated in captions.

## SUPPLEMENTARY MATERIAL

See the supplementary material for the figures and data tables.

## Data Availability

The data that support the findings of this study are openly available in NCBI's Gene Expression Omnibus (GEO, Ref. 111) and are accessible through GEO Series accession number GSE311002 (https://www.ncbi.nlm.nih.gov/geo/query/acc.cgi?acc=GSE311002).
